# Navigating the AI frontier: ethical considerations and best practices in microbial genomics research

**DOI:** 10.1099/mgen.0.001049

**Published:** 2023-06-07

**Authors:** Andrew J. Page, Niamh M. Tumelty, Samuel K. Sheppard

**Affiliations:** ^1^​ Theiagen Genomics, Highlands Ranch, Colorado, USA; ^2^​ University of East Anglia, Norfolk, UK; ^3^​ London School of Economics and Political Science, London, UK; ^4^​ Department of Biology, Ineos Oxford Institute, University of Oxford, Oxford, UK

**Keywords:** AI, ChatGPT, LLM, ethics

## Full-Text

Throughout history, writing has been a process of translating human thoughts into persistent text. The opening paragraphs of this editorial are no exception, written by the authors using English language semantic structures. However, quite soon, this manner of writing may attract a premium for its novelty, much as handmade crafts do today. The AI revolution has been ‘coming soon’ for decades, and has now arrived. Consistent with other information based professions, the research world is trying to catch-up with the new AI reality with new policies, procedures, ethics and education required.

Among the most disruptive AI technologies are deep learning models that generate human-like text based on prompts from users. These tools essentially allow AI computer programs, sometimes termed chatbots, to automatically write essays and manuscripts on a chosen subject. This has potentially far-reaching implications for scholarly publishing. For example, rapid information generation will exacerbate current publishing industry challenges around financial sustainability, shortage of peer reviewers and global equity for authors and readers. Publishing models will need to change in a world where it becomes feasible for an individual researcher to generate a manuscript for every experiment, potentially dozens of manuscripts per person per year and pre-existing issues of information overload with associated challenges around critical appraisal will be further intensified.

Some innovative models completely reimagine academic publishing, such as Octopus [https://www.octopus.ac]. These may be part of a solution by encouraging the generation of outputs that focus specifically on describing or reporting results of a particular experiment without additional narrative content that could be generated using large language models (chatbots). However, this does rather reduce the role of an editor or teacher, whose job it may be to select and emphasize significant or novel findings from a deluge of information.

AI in publishing is clearly a complex area with ongoing debate around the benefits and drawbacks. An interesting way to address this is to ask for input from an AI large language model (ChatGPT 4). What follows is an unedited generated output from the ‘mind’ of an AI. We invite readers to consider the content that has been generated. Specifically: levels of quality, comprehensiveness, textual accuracy and the AI’s reasoning of the ethical implications of its own existence. A companion podcast episode, also generated by ChatGPT 4, accompanies this article, with AI generated voices reading out the script. [https://soundcloud.com/research-pages/ep-13-navigating-the-ai-frontier-ethical-considerations-and-best-practices-in-academic-research]

## AI-generated editorial

Artificial intelligence (AI) has become an indispensable tool in the realm of academic research, offering a myriad of applications and opportunities for scientific advancement. In the field of microbial genomics, AI-powered techniques and tools have the potential to revolutionize the way researchers investigate and understand the complex genetic makeup of microorganisms. However, along with these opportunities come ethical considerations and challenges in implementing best practices for responsible AI use. This editorial explores the role of AI in microbial genomics research, the ethical considerations arising from the use of AI-generated content, best practices to ensure responsible use of AI, and the potential risks and benefits associated with AI-assisted research publishing.

## Role of AI in microbial genomics research

AI has the potential to contribute significantly to microbial genomics research by streamlining processes and enhancing data analysis. AI algorithms can facilitate the identification of genes, gene functions, and regulatory elements within microbial genomes. Furthermore, AI can help researchers make predictions about microbial behaviour, detect novel gene clusters, and even generate hypotheses for experimental validation. These capabilities can prove invaluable in accelerating the pace of discovery and improving our understanding of microorganisms and their interactions with their environment.

## Ethical considerations

Despite the promise of AI in microbial genomics research, the use of AI-generated content presents several ethical considerations that warrant attention:

1. Plagiarism and intellectual property rights: AI-generated content may inadvertently reproduce plagiarized material from its training data, raising concerns about originality and intellectual property rights. It is crucial for researchers to verify the originality of AI-generated content and properly cite sources to avoid plagiarism.
2. Bias in AI-generated content: AI models are trained on vast amounts of data, which might contain biases that can influence research findings. Researchers need to be aware of potential biases in AI-generated content and take steps to ensure their work is not influenced by them. This may include critically evaluating the outputs of AI and addressing any biassed information or perspectives.
3. Authorship and responsibility: the distinction between human and AI-generated content should be clear to avoid confusion about authorship and responsibility. Proper attribution is essential, and if AI-generated content is used in a publication, the specific AI tool should be cited to maintain transparency and give credit to the AI developers and the underlying data sources.

## Best practices for using AI-generated content in microbial genomics research

To ensure the responsible use of AI-generated content in microbial genomics research, researchers should adopt the following best practices:

1. Use AI as a supportive tool: AI should be considered a supportive tool rather than a replacement for human judgement and critical thinking. Researchers should use AI to enhance their research but not rely solely on AI-generated content.
2. Verify originality and proper citation: researchers should verify the originality of AI-generated content and ensure that any reused or rephrased content is properly cited to avoid plagiarism.
3. Acknowledge AI assistance: by citing the specific AI tool used in their research, researchers can maintain transparency and give credit to the AI developers and the underlying data sources.
4. Mitigate bias: researchers should be aware of potential biases in AI-generated content and take steps to minimize their impact. This might involve actively seeking out diverse perspectives, using unbiased datasets, or adjusting AI models to minimize bias.

## Risks and benefits of AI-assisted research publishing

The use of AI-generated content in microbial genomics research comes with potential risks and benefits:

## Risks

1. Plagiarism: as mentioned earlier, AI-generated content might inadvertently reproduce plagiarized material, which could lead to serious consequences for both the researcher and the institution.
2. Introduction of biases: AI models can inherit biases present in their training data, which could influence the research findings and compromise the integrity of the work. It’s crucial for researchers to be aware of these biases and take steps to minimize their impact.
3. Compromised research quality: over-reliance on AI-generated content may lead to compromised research quality if researchers don't critically evaluate AI-generated content and rely too heavily on it.
4. Misattribution of authorship and responsibility: when AI-generated content is used in research, it’s important to clearly differentiate between human and AI-generated content to avoid confusion about who is responsible for the research.

## Benefits

1. Increased efficiency: AI can help researchers quickly find relevant information, identify gaps in existing research, and even generate ideas and hypotheses. This enhanced efficiency can significantly benefit the microbial genomics research process.
2. Access to a broader range of information: by using AI to search through vast amounts of data, researchers can access knowledge that might have been difficult or time-consuming to find through traditional methods.
3. Assistance in generating ideas and hypotheses: AI-generated content can provide researchers with inspiration and help them overcome writer’s block or cognitive fatigue.
4. Promoting collaboration and interdisciplinary research: AI can help researchers discover connections between seemingly unrelated fields, fostering new collaborations and innovative research projects in microbial genomics.

## Conclusion

The use of AI-generated content in microbial genomics research holds great promise for enhancing the research process and accelerating scientific discovery. However, it also presents ethical challenges that need to be addressed. By adopting best practices, understanding potential risks and benefits, and maintaining a strong focus on human judgement and critical thinking, researchers can responsibly utilize AI-generated content in their work while minimizing potential risks associated with AI-assisted research publishing. As the field of microbial genomics continues to evolve and expand, the responsible use of AI will play an increasingly important role in shaping the future of scientific research.

